# Myocardial Function Assessment After Cardioversion in Persistent Atrial Fibrillation: Current Evidence and Future Directions

**DOI:** 10.3390/diagnostics16162548

**Published:** 2026-08-12

**Authors:** Emma Sokolova, Ainārs Rudzītis, Oskars Kalējs

**Affiliations:** 1 Department of Doctoral Studies, Rīga Stradiņš University, LV-1007 Riga, Latvia; 2 Department of Internal Diseases, Faculty of Medicine, Rīga Stradiņš University, LV-1007 Riga, Latvia; 3 Centre of Clinical Diagnostics, Rīga East Clinical University Hospital, LV-1079 Riga, Latvia; 4 Latvian Centre of Cardiology, Pauls Stradiņš Clinical University Hospital, LV-1002 Riga, Latvia

**Keywords:** atrial fibrillation, electrical cardioversion, myocardial function, myocardial recovery, reverse remodeling, global longitudinal strain, echocardiography, cardiac biomarkers, rhythm control, personalized medicine

## Abstract

Persistent atrial fibrillation (AF) is associated with complex structural, electrical, and functional myocardial remodeling that contributes to impaired cardiac performance, symptom burden, and adverse cardiovascular outcomes. Although restoration of sinus rhythm through electrical cardioversion remains a cornerstone of rhythm-control therapy, treatment success is traditionally defined by rhythm maintenance and recurrence rates, while myocardial functional recovery receives substantially less attention. This review aims to summarize current evidence regarding myocardial dysfunction in persistent AF, evaluate available approaches for assessing myocardial recovery after cardioversion, and explore future directions for function-guided rhythm-control strategies. A narrative review of contemporary literature was performed, focusing on studies published in major cardiovascular journals and current international guidelines. Evidence regarding myocardial remodeling, reverse remodeling after restoration of sinus rhythm, echocardiographic assessment, myocardial strain imaging, biomarker dynamics, and post-cardioversion management strategies was synthesized and critically evaluated. Persistent AF induces multidimensional myocardial dysfunction through tachycardia-mediated injury, structural remodeling, neurohormonal activation, inflammation, and fibrosis. Emerging evidence suggests that restoration of sinus rhythm may initiate a process of reverse remodeling characterized by improvements in left ventricular systolic function, global longitudinal strain, atrial mechanical performance, and biomarker profiles. Advanced echocardiographic techniques and serial biomarker assessment provide valuable opportunities to quantify myocardial recovery beyond conventional rhythm-based endpoints. However, current clinical practice and guideline-directed management remain predominantly focused on rhythm outcomes, while functional recovery is not routinely incorporated into therapeutic decision-making. Based on available evidence, a conceptual framework termed the “Post-Cardioversion Functional Window” is proposed to describe a period of potentially enhanced myocardial recovery during which systematic functional assessment may facilitate individualized management and future risk stratification. Myocardial recovery after restoration of sinus rhythm represents an underrecognized component of contemporary AF management. Integration of echocardiographic, biomarker-based, and clinical functional assessment may improve understanding of post-cardioversion remodeling and support the development of personalized rhythm-control strategies. Future prospective studies are needed to validate myocardial recovery endpoints and determine their role in guiding therapeutic decisions after cardioversion.

## 1. Introduction

Atrial fibrillation (AF) is the most common sustained cardiac arrhythmia and represents a major clinical and public health challenge. Its prevalence is increasing globally, driven by population ageing, improved detection, and the rising burden of cardiovascular risk factors and comorbidities [1,2]. AF is associated with stroke, heart failure, recurrent hospitalization, reduced quality of life, cognitive decline, and premature mortality, making its management a priority for contemporary cardiovascular care [1,2,3].

Recent international guidelines have emphasized the need for comprehensive, structured, and individualized AF management. The 2024 European Society of Cardiology (ESC) Guidelines introduced the AF-CARE framework, which highlights comorbidity management, stroke prevention, symptom control, rhythm assessment, and continuous reassessment across the patient pathway [1]. Similarly, the 2023 ACC/AHA/ACCP/HRS Guideline underlines the importance of early diagnosis, risk-factor modification, and individualized rhythm-control decisions [3]. Within this evolving paradigm, rhythm control is increasingly viewed not only as a strategy for symptom relief but also as a potential means of improving long-term cardiovascular outcomes in selected patients [4].

Electrical cardioversion remains one of the most frequently used rhythm-control interventions in patients with persistent AF. In routine clinical practice, the success of cardioversion is usually defined by immediate restoration of sinus rhythm and subsequent maintenance of rhythm over time. However, AF is not merely an electrical disorder. Persistent AF is associated with complex electrical, structural, mechanical, and neurohormonal remodeling involving both atrial and ventricular myocardium [5,6]. Prolonged exposure to irregular rhythm, tachycardia, loss of coordinated atrial contraction, and comorbidity-related myocardial stress may contribute to impaired cardiac performance and progressive myocardial dysfunction [5,6,7,8].

The concept of AF-mediated or arrhythmia-induced cardiomyopathy has gained increasing attention, reflecting the potentially reversible relationship between sustained arrhythmia and myocardial dysfunction [8,9,10]. Although tachycardia is an important mechanism, irregular ventricular activation, loss of atrioventricular synchrony, altered calcium handling, neurohormonal activation, and atrial mechanical failure may also contribute to ventricular impairment and heart failure progression [8,9,10]. Importantly, myocardial dysfunction may be present even when left ventricular ejection fraction (LVEF) is preserved, indicating the need for more sensitive tools to characterize functional impairment and recovery [11].

Restoration of sinus rhythm may initiate a process of reverse remodeling, with potential improvements in ventricular systolic function, atrial mechanical performance, myocardial deformation parameters, and biomarker profiles [9,12,13]. Prospective data in arrhythmia-induced cardiomyopathy suggest that recovery after rhythm restoration may occur over several months, supporting the concept that rhythm-control success should not be assessed only by rhythm status but also by functional cardiac recovery [12]. Similarly, biomarker studies after electrical cardioversion have shown that changes in NT-proBNP and other circulating markers may reflect rhythm status and myocardial adaptation after the restoration of sinus rhythm [13].

Advances in echocardiography provide new opportunities to evaluate myocardial recovery beyond conventional LVEF assessment. Global longitudinal strain (GLS) is a sensitive marker of myocardial dysfunction and has demonstrated prognostic value in patients with heart failure, including those with preserved ejection fraction [11]. In patients with AF, atrial strain imaging may provide additional information regarding atrial mechanical remodeling and recurrence risk. Recent evidence suggests that both left and right atrial strain parameters may improve the prediction of AF recurrence after electrical cardioversion, highlighting the potential role of functional imaging in post-cardioversion risk stratification [14].

Despite these developments, myocardial functional recovery after cardioversion remains insufficiently integrated into contemporary AF management. Current clinical pathways and trial endpoints continue to focus predominantly on rhythm maintenance, AF recurrence, hospitalization, and major cardiovascular events, while the trajectory and clinical significance of myocardial recovery are less consistently evaluated. This creates an important knowledge gap: should successful rhythm control be defined only by restoration and maintenance of sinus rhythm, or should it also include objective evidence of myocardial functional improvement?

This narrative review aims to summarize current evidence regarding myocardial dysfunction in persistent AF and myocardial recovery following the restoration of sinus rhythm. Particular attention is given to echocardiographic assessment, myocardial strain imaging, biomarker-based evaluation, and the diagnostic and prognostic value of functional recovery after cardioversion. Furthermore, we propose the concept of a “Post-Cardioversion Functional Window”, defined as a period of potentially enhanced myocardial recovery during which systematic functional assessment may support personalized rhythm-control management and inform future prospective research.

### Literature Search and AI-Assisted Figure Development

This narrative review was based on a critical evaluation and synthesis of the published literature. Artificial intelligence (ChatGPT, OpenAI, GPT-5.5) was used to generate conceptual visualization of the schematic figures illustrating the theoretical frameworks presented in this review. The scientific concepts, literature interpretation, figure design, terminology, and all final scientific content were independently developed, critically reviewed, substantially revised, verified against the published literature, and approved by the authors. Artificial intelligence (AI) was not used for data generation, data analysis, interpretation of scientific evidence, or formulation of scientific conclusions.

All AI-generated figures underwent substantial manual revision by the authors. Medical terminology, scientific content, figure structure, graphical elements, captions, terminology, and clinical interpretation were independently reviewed, modified, and verified against the published literature. The final figures were generated using ChatGPT (OpenAI, GPT-5.5) based on author-developed scientific concepts and detailed prompts. They subsequently underwent substantial manual scientific revision, including modification of terminology, graphical layout, annotations, and explanatory elements, and were independently verified against the cited literature. The authors assume full responsibility for the scientific accuracy of all figures.

## 2. Myocardial Dysfunction in Persistent AF

### 2.1. AF-Mediated Cardiomyopathy and Tachycardia-Induced Dysfunction

Persistent atrial fibrillation is increasingly recognized as a condition capable of directly contributing to myocardial dysfunction. Although AF frequently coexists with pre-existing cardiovascular disease, accumulating evidence supports the concept that sustained arrhythmia itself may induce a potentially reversible form of cardiomyopathy, commonly referred to as arrhythmia-induced cardiomyopathy (AiCM) or AF-mediated cardiomyopathy [15,16].

Historically, myocardial dysfunction associated with AF was primarily attributed to tachycardia-induced cardiomyopathy. However, contemporary evidence suggests a considerably more complex pathophysiological process. In addition to persistent tachycardia, irregular ventricular activation, loss of atrioventricular synchrony, impaired atrial mechanical function, altered calcium handling, autonomic imbalance, and neurohormonal activation all contribute to myocardial impairment [15,17]. Experimental and clinical studies indicate that AF-related ventricular dysfunction may occur even in the absence of sustained rapid ventricular rates, suggesting that rhythm irregularity itself may represent an independent pathological mechanism [18].

Recent state-of-the-art reviews have emphasized that AF-mediated cardiomyopathy should be viewed as a distinct clinical entity within the broader spectrum of arrhythmia-induced cardiomyopathies [15]. The diagnosis is often challenging because AF frequently develops in patients with underlying structural heart disease, making it difficult to determine the relative contribution of the arrhythmia to ventricular dysfunction. Nevertheless, the hallmark characteristic of AF-mediated cardiomyopathy remains its partial or complete reversibility following successful rhythm control and restoration of sinus rhythm [15,16].

The prevalence of arrhythmia-induced cardiomyopathy among patients with AF remains incompletely defined. Prospective investigations suggest that a substantial proportion of patients presenting with otherwise unexplained left ventricular systolic dysfunction and concomitant atrial tachyarrhythmias demonstrate significant recovery of ventricular function after rhythm correction [19]. Recovery may occur over several weeks to months and appears to depend on arrhythmia duration, ventricular rate burden, baseline myocardial substrate, and the effectiveness of rhythm-control interventions [19].

Importantly, myocardial dysfunction in AF is not limited to reductions in left ventricular ejection fraction. Subclinical impairment of myocardial mechanics may precede overt systolic dysfunction and can be detected using advanced imaging modalities, particularly myocardial strain imaging. This observation has important clinical implications because patients with preserved ejection fraction may nevertheless experience significant myocardial dysfunction that remains undetected by conventional echocardiographic assessment [11].

These findings support the concept that successful AF management should extend beyond rhythm restoration alone and include assessment of myocardial recovery. Understanding the mechanisms underlying AF-mediated myocardial dysfunction provides the foundation for evaluating reverse remodeling after cardioversion and for identifying patients who may derive the greatest benefit from personalized rhythm-control strategies.

### 2.2. Structural and Mechanical Remodeling

Persistent AF promotes progressive structural remodeling of both atrial and ventricular myocardium. Structural remodeling is characterized by myocyte hypertrophy, extracellular matrix expansion, fibroblast activation, and progressive myocardial fibrosis, resulting in alterations of chamber geometry and mechanical performance [20,21].

Within the atria, fibrosis represents one of the most important determinants of AF perpetuation. Histological and imaging studies have demonstrated that prolonged AF is associated with increasing degrees of atrial fibrosis, loss of myocardial fiber organization, and impaired atrial contractile function [20]. These changes contribute to electrical heterogeneity, impaired conduction, and increased susceptibility to recurrent arrhythmia.

Mechanical remodeling develops in parallel with structural alterations. Even in the presence of preserved left ventricular ejection fraction, patients with persistent AF frequently demonstrate impaired myocardial deformation, reduced atrial reservoir function, and abnormalities of ventricular filling dynamics [22]. Advanced echocardiographic techniques have shown that atrial strain parameters may decline before overt structural changes become evident, supporting their role as early markers of atrial dysfunction [23].

The left atrium plays a particularly important role in the pathophysiology of AF. Progressive enlargement and impairment of atrial mechanics contribute not only to arrhythmia maintenance but also to thromboembolic risk, symptom burden, and heart failure progression. Recent studies suggest that left atrial strain may provide incremental prognostic information beyond conventional measurements such as left atrial diameter or volume [23,24].

Importantly, structural remodeling is not necessarily irreversible. Restoration of sinus rhythm may initiate varying degrees of reverse remodeling, particularly when intervention occurs before extensive fibrosis has developed. This concept forms the basis for contemporary rhythm-control strategies and supports the need for serial assessment of myocardial function after cardioversion.

The mechanisms contributing to myocardial dysfunction in persistent AF are multifactorial and involve electrical, structural, mechanical, neurohormonal, and inflammatory pathways. A summary of the principal mechanisms and their clinical consequences is presented in Table 1.

**Figure 1:**
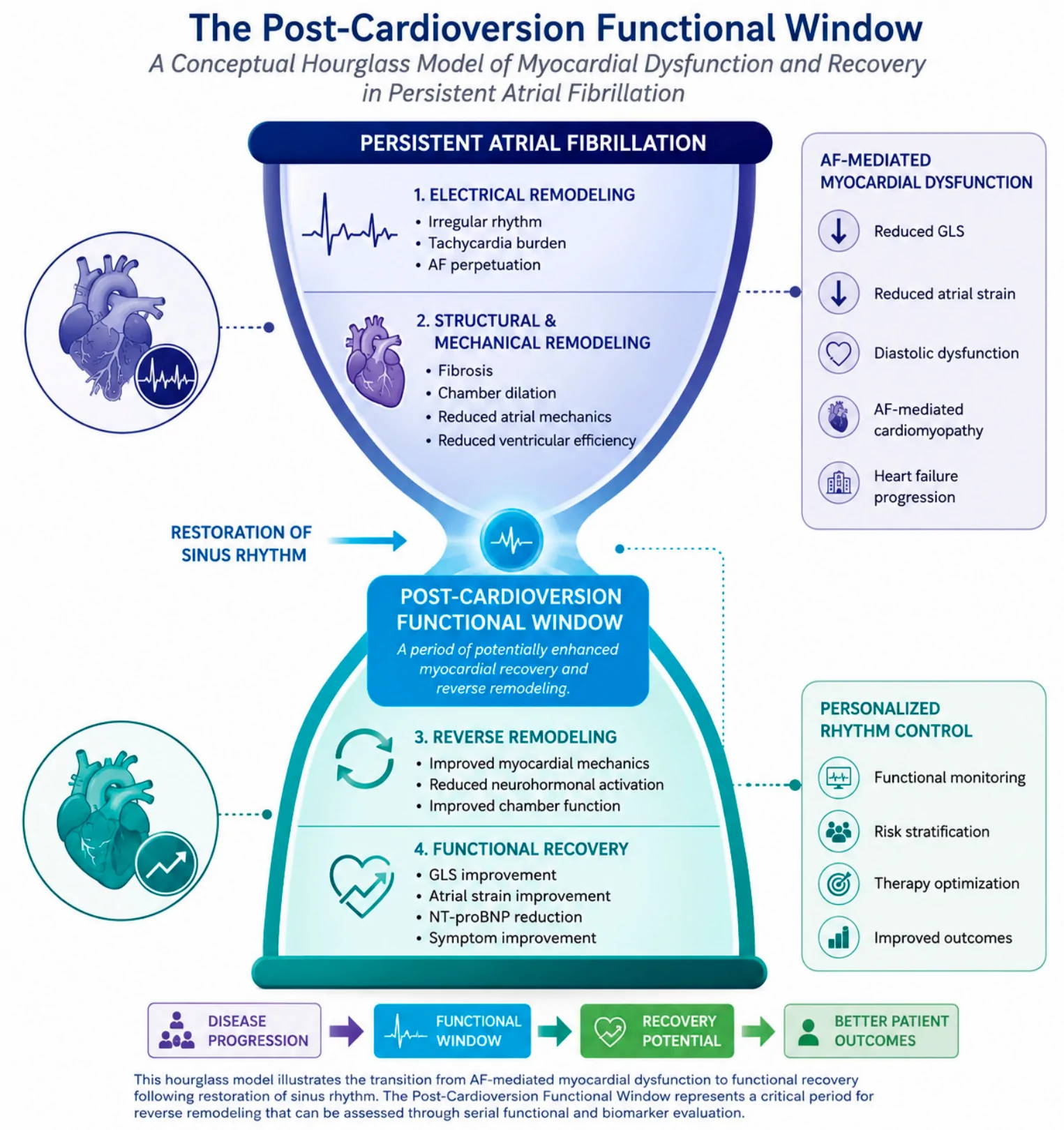
From persistent atrial fibrillation to myocardial recovery: a conceptual framework of remodeling, dysfunction, and therapeutic opportunity. Persistent atrial fibrillation promotes electrical, structural, and mechanical remodeling that may result in myocardial dysfunction. Restoration of sinus rhythm may initiate reverse remodeling and functional improvement during the proposed Post-Cardioversion Functional Window. Abbreviations: AF, atrial fibrillation; GLS, global longitudinal strain; NT-proBNP, N-terminal pro-B-type natriuretic peptide.

The progression from persistent AF to myocardial dysfunction involves a complex interaction of electrical, structural, and mechanical remodeling processes. These mechanisms, together with the conceptual framework of a Post-Cardioversion Functional Window, are summarized in Figure 1.

### 2.3. Neurohormonal Activation, Inflammation, and Fibrosis

Beyond electrical and mechanical abnormalities, persistent AF is characterized by profound neurohormonal, inflammatory, and fibrotic remodeling. These biological processes contribute not only to arrhythmia maintenance but also to progressive myocardial dysfunction and adverse cardiovascular outcomes [25,26].

Activation of the renin–angiotensin–aldosterone system (RAAS) and sympathetic nervous system is frequently observed in patients with AF and heart failure. Experimental and clinical studies suggest that sustained neurohormonal activation promotes myocardial hypertrophy, oxidative stress, fibroblast proliferation, and extracellular matrix deposition, thereby facilitating both atrial and ventricular remodeling [25,27]. In addition, increased catecholamine activity may contribute to electrical instability and further perpetuate arrhythmogenesis.

Inflammation has emerged as another important contributor to AF pathophysiology. Elevated circulating levels of inflammatory biomarkers, including C-reactive protein, interleukin-6, tumor necrosis factor-α, and other cytokines, have been associated with AF occurrence, persistence, and recurrence [26,28]. Inflammatory signaling pathways promote structural remodeling through the activation of fibroblasts and stimulation of collagen synthesis, resulting in progressive myocardial fibrosis.

Myocardial fibrosis represents a final common pathway linking electrical, structural, and inflammatory remodeling. Fibrotic changes alter myocardial architecture, impair conduction, reduce myocardial compliance, and contribute to mechanical dysfunction [29]. Importantly, the extent of fibrosis has been associated with rhythm-control outcomes, including recurrence after cardioversion and catheter ablation [29,30]. These observations suggest that fibrosis may serve not only as a marker of disease progression but also as a determinant of myocardial recovery potential.

Recent evidence indicates that biological remodeling may remain partially reversible, particularly during earlier stages of disease. Restoration of sinus rhythm has been associated with reductions in neurohormonal activation, improvements in natriuretic peptide profiles, and favorable changes in myocardial mechanics [13,15]. However, the degree of reversibility appears to depend on the duration of AF, burden of comorbidities, and extent of pre-existing fibrosis [29].

Taken together, neurohormonal activation, inflammation, and fibrosis constitute critical biological mechanisms linking persistent AF to myocardial dysfunction. Understanding these pathways is essential for identifying patients most likely to benefit from rhythm restoration and for interpreting markers of myocardial recovery during the post-cardioversion period.

The principal biological mechanisms involved in AF-related myocardial dysfunction and their potential implications for functional recovery assessment are summarized in Table 2.

Collectively, the evidence reviewed in this section suggests that myocardial dysfunction in persistent AF extends far beyond rhythm disturbance alone. Electrical instability, structural remodeling, mechanical impairment, neurohormonal activation, inflammation, and fibrosis interact to form a complex and dynamic disease process that affects both atrial and ventricular myocardium. Importantly, many of these abnormalities may develop before overt reductions in left ventricular ejection fraction become apparent, highlighting the limitations of relying exclusively on conventional measures of cardiac function.

Recent advances in imaging and biomarker assessment have improved our understanding of AF-related myocardial dysfunction and have revealed substantial heterogeneity among patients with persistent AF. While some individuals demonstrate advanced remodeling and limited reversibility, others exhibit significant recovery of myocardial function following the restoration of sinus rhythm. This variability suggests that myocardial dysfunction in AF should be viewed not as a static phenomenon but as a potentially modifiable process influenced by disease duration, comorbidity burden, underlying myocardial substrate, and treatment timing [12,15,19,29].

Despite increasing recognition of AF-mediated cardiomyopathy and reverse remodeling, the recovery phase following the restoration of sinus rhythm remains insufficiently characterized. Current rhythm-control strategies predominantly focus on arrhythmia recurrence and rhythm maintenance, whereas the extent, timing, and clinical significance of myocardial recovery are less frequently assessed. As a result, an important opportunity may be missed to identify patients who derive the greatest functional benefit from successful rhythm restoration.

Understanding the mechanisms and trajectory of myocardial recovery after cardioversion therefore represents a critical next step in the evolution of personalized rhythm-control management. The following section reviews current evidence regarding reverse remodeling, the recovery of myocardial mechanics, and functional improvement following the restoration of sinus rhythm, with particular emphasis on imaging and biomarker-based assessment.

## 3. Myocardial Recovery After Restoration of Sinus Rhythm

### 3.1. Reverse Remodeling After Restoration of Sinus Rhythm

The restoration of sinus rhythm represents more than the correction of an electrical abnormality. Increasing evidence suggests that successful rhythm restoration may initiate a process of reverse cardiac remodeling involving structural, mechanical, and neurohormonal recovery. This concept has gained relevance in patients with persistent AF, where prolonged exposure to arrhythmia contributes to progressive myocardial dysfunction and AF-mediated cardiomyopathy [31,32].

Reverse remodeling refers to the partial or complete reversal of pathological changes that develop during AF. These changes may include improvements in myocardial contractility, chamber geometry, atrial mechanical function, neurohormonal activation, and myocardial deformation parameters [15,31]. Although the degree of recovery varies among individuals, multiple studies have demonstrated that the restoration of sinus rhythm may be associated with measurable improvements in cardiac structure and function over time.

The biological basis of reverse remodeling is multifactorial. The restoration of coordinated atrioventricular activation improves ventricular filling and cardiac output, while the elimination of persistent tachyarrhythmia reduces myocardial energy expenditure and abnormal calcium handling [15,17]. In parallel, reductions in sympathetic activation and neurohormonal stimulation may contribute to favorable alterations in myocardial metabolism and extracellular matrix remodeling [25,26]. Collectively, these mechanisms create conditions that may facilitate myocardial recovery following successful rhythm control.

Evidence supporting reverse remodeling has emerged from studies evaluating both cardioversion and catheter ablation. Improvements in left ventricular systolic function, reductions in chamber volumes, and recovery of atrial mechanical function have been reported following the restoration of sinus rhythm, particularly among patients with AF-mediated cardiomyopathy [32,33,34]. Notably, recovery often continues for several weeks or months after rhythm restoration, suggesting that myocardial adaptation is a dynamic process rather than an immediate consequence of successful cardioversion.

The extent of reverse remodeling appears to depend on multiple factors, including AF duration, baseline myocardial function, comorbidity burden, age, and the degree of pre-existing fibrosis [29,35]. Patients with advanced structural remodeling may demonstrate limited recovery despite successful rhythm control, whereas individuals with earlier-stage disease frequently exhibit substantial functional improvement. These observations support the concept that myocardial recovery represents a spectrum rather than a binary outcome.

Importantly, current clinical practice rarely evaluates reverse remodeling as a therapeutic endpoint. Rhythm-control success is generally defined by the maintenance of sinus rhythm, while objective measures of myocardial recovery are assessed inconsistently. However, the growing recognition of AF-mediated cardiomyopathy and reversible myocardial dysfunction suggests that the assessment of reverse remodeling may provide clinically meaningful information beyond rhythm status alone.

Taken together, the available evidence indicates that the restoration of sinus rhythm may initiate a period of active myocardial adaptation characterized by structural, mechanical, and biological recovery. This evolving understanding provides a foundation for the systematic assessment of myocardial function following cardioversion and raises the possibility that recovery trajectories may offer additional prognostic and therapeutic value beyond conventional rhythm-based outcomes.

**Figure 2:**
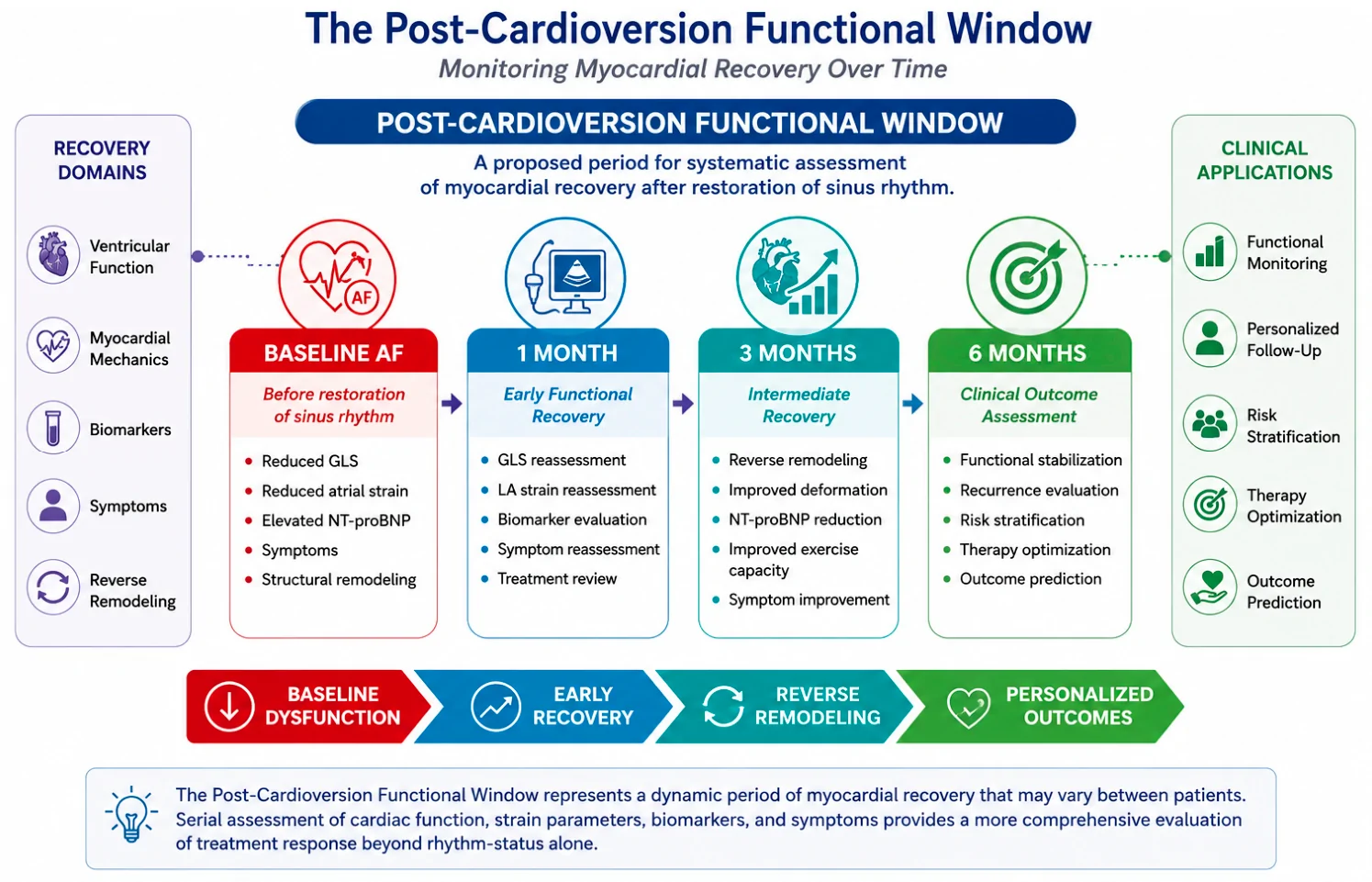
The Post-Cardioversion Functional Window: temporal evolution of myocardial recovery after restoration of sinus rhythm. The proposed framework illustrates a dynamic period in which serial assessment may characterize myocardial deformation, atrial mechanics, biomarker profiles, symptoms, and reverse remodeling. Abbreviations: AF, atrial fibrillation; GLS, global longitudinal strain; LA, left atrial; NT-proBNP, N-terminal pro-B-type natriuretic peptide.

The temporal evolution of myocardial recovery following the restoration of sinus rhythm is illustrated in Figure 2.

### 3.2. Recovery of Left Ventricular Function

One of the most clinically relevant manifestations of myocardial recovery following the restoration of sinus rhythm is improvement in left ventricular function. While AF has traditionally been regarded as a supraventricular arrhythmia, growing evidence demonstrates that persistent AF may exert substantial adverse effects on ventricular performance, even in the absence of overt structural heart disease [15,31].

The relationship between AF and ventricular dysfunction is bidirectional. Left ventricular dysfunction predisposes patients to AF development, while sustained AF may further impair ventricular performance through irregular ventricular activation, loss of atrioventricular synchrony, reduced diastolic filling, neurohormonal activation, and tachycardia-mediated myocardial injury [15,17,32]. Consequently, the restoration of sinus rhythm may initiate measurable improvements in ventricular function by addressing several of these mechanisms simultaneously.

Studies evaluating patients with AF-mediated cardiomyopathy have consistently demonstrated significant recovery of left ventricular systolic function following successful rhythm control [32,34,36]. In many patients, improvements in left ventricular ejection fraction (LVEF) become evident within weeks after the restoration of sinus rhythm and may continue for several months. Importantly, the magnitude of recovery appears greatest among patients with previously unrecognized arrhythmia-induced myocardial dysfunction, suggesting that AF itself may represent a reversible cause of heart failure in selected individuals [32].

The CASTLE-AF trial and subsequent investigations further strengthened the concept that the restoration and maintenance of sinus rhythm may improve ventricular function and clinical outcomes in patients with concomitant AF and heart failure [33,37]. Although many studies have focused on catheter ablation, the biological mechanisms underlying recovery are likely similar following successful electrical cardioversion, particularly when sinus rhythm is maintained during follow-up.

Nevertheless, recovery trajectories are highly heterogeneous. Some patients demonstrate rapid normalization of ventricular function, whereas others experience only modest improvement despite successful rhythm restoration [31,35]. The degree of reversibility appears to depend on multiple factors, including AF duration, baseline ventricular dysfunction, age, comorbidity burden, and the extent of myocardial fibrosis. These findings highlight the importance of individualized assessment rather than assuming uniform recovery across all patients.

Importantly, a reliance on LVEF alone may underestimate the true extent of myocardial dysfunction and recovery. Conventional measurements often fail to detect subtle abnormalities in myocardial mechanics, particularly among patients with preserved ejection fraction. Consequently, newer imaging techniques such as global longitudinal strain (GLS) have attracted increasing attention as more sensitive indicators of myocardial function and reverse remodeling [11].

From a clinical perspective, the assessment of ventricular recovery may provide information beyond simple rhythm status. Patients demonstrating significant functional improvement following cardioversion may represent a distinct phenotype characterized by greater myocardial reversibility and potentially more favorable long-term outcomes. Conversely, limited recovery despite the maintenance of sinus rhythm may indicate advanced myocardial disease requiring additional therapeutic strategies.

Taken together, the available evidence suggests that recovery of the left ventricular function constitutes a central component of post-cardioversion myocardial adaptation. However, conventional echocardiographic parameters alone may not fully capture the complexity of this process. This limitation has stimulated growing interest in myocardial deformation imaging and strain-based assessment as more sensitive tools for evaluating functional recovery after restoration of sinus rhythm.

**Figure 3:**
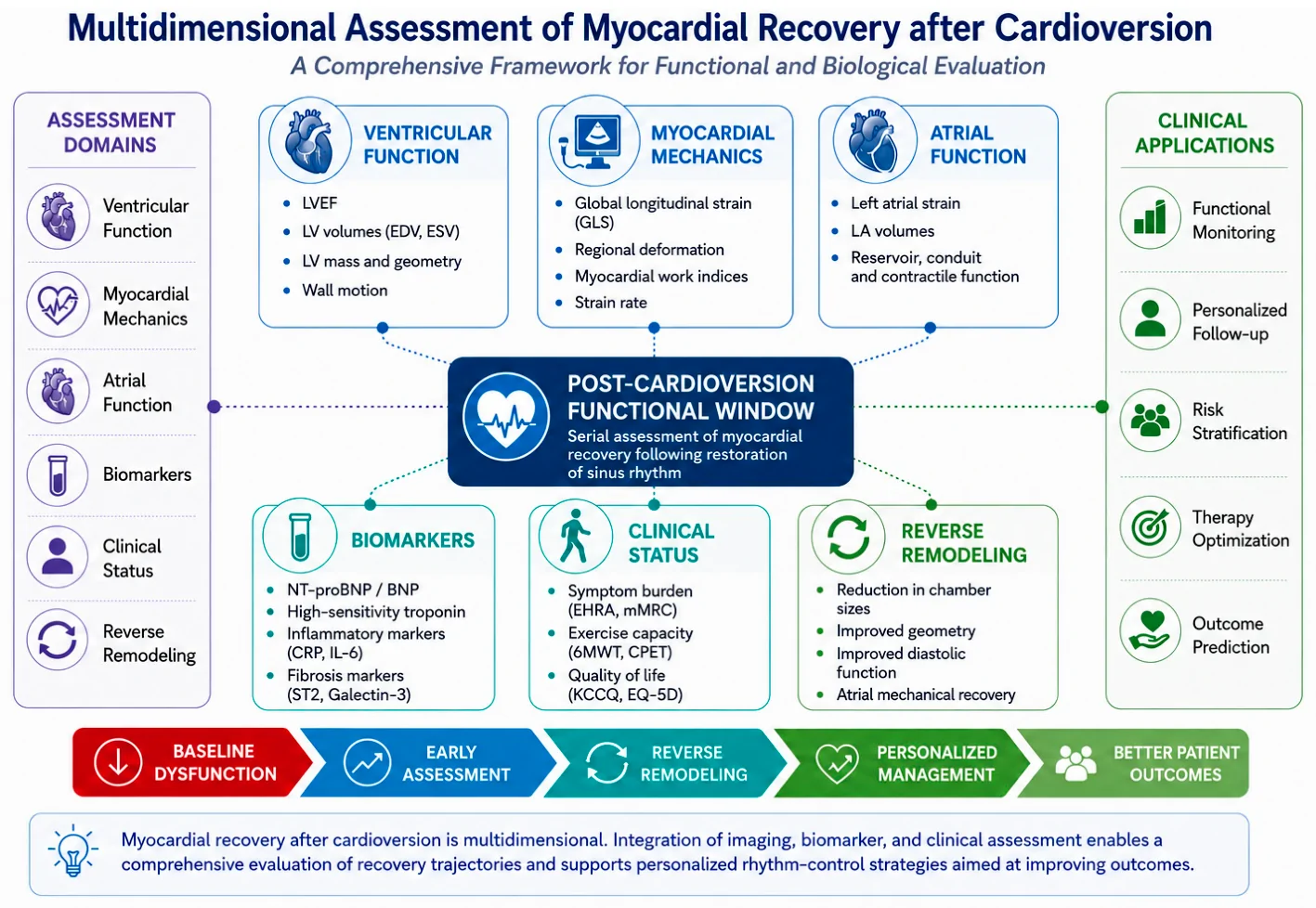
Multidimensional assessment of myocardial recovery after cardioversion. Recovery following restoration of sinus rhythm extends beyond conventional measures of ventricular systolic function. A comprehensive assessment framework should integrate ventricular function, myocardial deformation imaging, biomarker dynamics, symptoms, exercise capacity, and evidence of reverse remodeling. Such a multidimensional approach may facilitate personalized rhythm-control management and improve characterization of recovery trajectories during the post-cardioversion period. Abbreviations: 6MWT, 6-min walk test; BNP, B-type natriuretic peptide; CPET, cardiopulmonary exercise testing; CRP, C-reactive protein; EDV, end-diastolic volume; EHRA, European Heart Rhythm Association symptom classification; EQ-5D, EuroQol 5-Dimension questionnaire; ESV, end-systolic volume; GLS, global longitudinal strain; IL-6, interleukin-6; KCCQ, Kansas City Cardiomyopathy Questionnaire; LA, left atrial; LV, left ventricular; LVEF, left ventricular ejection fraction; mMRC, modified Medical Research Council dyspnea scale; NT-proBNP, N-terminal pro-B-type natriuretic peptide; ST2, suppression of tumorigenicity 2.

Conventional echocardiographic assessment and advanced myocardial deformation imaging provide complementary information regarding post-cardioversion recovery. The proposed multidimensional approach to ventricular functional assessment is summarized in Figure 3.

### 3.3. Recovery of Myocardial Mechanics: Global Longitudinal Strain and Strain Imaging

Although the left ventricular ejection fraction remains the most widely used parameter for assessing cardiac function, it may not adequately capture subtle myocardial abnormalities associated with persistent AF. Increasing evidence suggests that myocardial deformation imaging, particularly global longitudinal strain (GLS) and atrial strain assessment, provides a more sensitive evaluation of myocardial dysfunction and recovery following the restoration of sinus rhythm [11,38].

Global longitudinal strain reflects longitudinal myocardial fiber shortening and has emerged as an important marker of subclinical ventricular dysfunction. Numerous studies have demonstrated that impaired GLS may be present despite preserved LVEF, indicating that conventional systolic function measurements may underestimate the true burden of myocardial dysfunction [11,39]. In patients with AF, abnormalities in GLS have been associated with symptom burden, exercise intolerance, heart failure progression, and adverse cardiovascular outcomes [39].

Following the restoration of sinus rhythm, improvements in GLS frequently occur before substantial changes in LVEF become apparent. This observation suggests that myocardial deformation imaging may detect early stages of reverse remodeling and functional recovery [40]. Improvements in GLS have been reported after both electrical cardioversion and catheter ablation, supporting the concept that the restoration of coordinated myocardial activation may facilitate the recovery of ventricular mechanics independent of changes in global systolic function [40,41].

Atrial strain imaging provides complementary information regarding atrial mechanical recovery. AF is associated with progressive impairment of the atrial reservoir, conduit, and contractile function, reflecting underlying structural remodeling and fibrosis [23,42]. Even after successful cardioversion, the recovery of atrial mechanics may occur gradually and may lag the restoration of electrical sinus rhythm. Consequently, atrial strain assessment offers a unique opportunity to evaluate functional recovery of the atrial myocardium rather than rhythm status alone.

Several studies have demonstrated that left atrial strain parameters are associated with the maintenance of sinus rhythm, recurrence risk, and overall cardiovascular prognosis [14,42,43]. Patients exhibiting greater improvement in atrial strain following the restoration of sinus rhythm appear more likely to maintain rhythm stability and demonstrate favorable remodeling trajectories. These findings suggest that atrial strain may serve both as a marker of recovery and as a prognostic indicator of long-term rhythm-control success.

Importantly, myocardial recovery is unlikely to be adequately described by a single parameter. Rather, recovery should be viewed as a multidimensional process involving ventricular mechanics, atrial function, chamber remodeling, biomarker dynamics, and clinical improvement. The integration of GLS and atrial strain into post-cardioversion assessment may therefore provide a more comprehensive evaluation of myocardial adaptation than conventional echocardiographic parameters alone.

From a translational perspective, strain imaging may represent one of the most promising tools for characterizing the Post-Cardioversion Functional Window. Serial assessment of GLS and atrial strain could potentially identify patients demonstrating active reverse remodeling, distinguish reversible myocardial dysfunction from advanced myocardial disease, and support more individualized rhythm-control strategies. However, prospective studies specifically evaluating strain-guided management after cardioversion remain limited and represent an important area for future investigation.

The framework presented in Figure 3 highlights the multidimensional nature of myocardial recovery following the restoration of sinus rhythm. Although improvement in left ventricular ejection fraction remains an important marker of functional recovery, growing evidence suggests that recovery after cardioversion encompasses multiple interrelated domains, including myocardial mechanics, atrial function, biomarker dynamics, symptom burden, exercise capacity, and structural remodeling. The evaluation of a single parameter may therefore provide only a partial representation of the overall recovery process.

Importantly, recovery trajectories appear highly heterogeneous among patients with persistent AF. While some individuals demonstrate substantial improvements across several domains, others experience limited functional recovery despite the successful maintenance of sinus rhythm. This variability supports the concept that post-cardioversion recovery should be viewed as a spectrum rather than a binary outcome and highlights the need for more sensitive methods capable of characterizing individual recovery patterns.

Among the available assessment tools, myocardial deformation imaging has emerged as one of the most promising approaches for detecting early functional improvement. Global longitudinal strain and atrial strain may identify subtle changes in myocardial performance before conventional echocardiographic parameters become abnormal or demonstrate measurable improvement. Consequently, strain imaging may provide unique insights into the biological processes occurring during the conceptual framework Post-Cardioversion Functional Window and may facilitate a more precise characterization of myocardial recovery.

The following section focuses on the role of myocardial deformation imaging and examines current evidence regarding the recovery of ventricular and atrial mechanics after the restoration of sinus rhythm.

### 3.4. Multimodality Cardiac Imaging in the Assessment of Post-Cardioversion Recovery

Echocardiography should remain the first-line modality for serial assessment within the proposed framework. It is widely available, relatively inexpensive, free of ionizing radiation, and suitable for repeated evaluation of chamber dimensions, left ventricular ejection fraction, diastolic function, global longitudinal strain, and atrial mechanics. These characteristics make echocardiography the most practical method for establishing recovery trajectories at predefined follow-up intervals. Its limitations include operator and acoustic-window dependence, inter-vendor variability of deformation measurements, and reduced reproducibility when measurements are obtained during an irregular rhythm [11,38,39,40,41,42,43,44].

Cardiac magnetic resonance (CMR) provides complementary information when more precise phenotyping is required. In addition to highly reproducible ventricular volumes and systolic function, CMR enables tissue characterization using late gadolinium enhancement, native T1 mapping, and extracellular volume. These techniques may help distinguish potentially reversible arrhythmia-mediated dysfunction from a fixed fibrotic substrate and may help explain limited recovery despite the maintenance of sinus rhythm. The absence of late gadolinium enhancement has been associated with greater ventricular recovery after rhythm restoration, while serial CMR studies have demonstrated regression of diffuse fibrosis in selected patients [19,30,34,41,44]. However, cost, availability, scan duration, device- and patient-related constraints, and the need for gadolinium in fibrosis assessment limit its suitability for frequent routine follow-up.

Cardiac computed tomography (CCT) offers excellent spatial resolution and is particularly useful for coronary assessment, pulmonary vein and left atrial anatomy, left atrial appendage evaluation, and procedural planning. It may therefore clarify important structural or ischemic contributors to an unfavorable recovery trajectory. Nevertheless, radiation exposure, iodinated contrast, and less established myocardial tissue characterization make CCT less appropriate than echocardiography or CMR for repeated functional assessment [44]. Positron emission tomography and single-photon emission computed tomography may have targeted roles when ischemia, viability, or inflammatory cardiomyopathy is suspected, but they are not proposed as routine post-cardioversion surveillance tools [1,3].

A tiered multimodality strategy is therefore most appropriate: echocardiography for baseline and serial monitoring; CMR for myocardial substrate characterization, discordant findings, or unexplained incomplete recovery; and CCT or nuclear imaging for specific anatomical, coronary, ischemic, or inflammatory indications. This approach preserves the feasibility of the framework while allowing deeper phenotyping when clinically justified.

### 3.5. Biomarker Dynamics During Recovery

Biomarkers have become an increasingly important component of cardiovascular risk assessment and may provide valuable insights into myocardial recovery following the restoration of sinus rhythm. Unlike imaging parameters, which primarily characterize structural and functional adaptation, circulating biomarkers may reflect underlying biological processes such as myocardial stress, neurohormonal activation, inflammation, and fibrosis [13,45].

Among currently available biomarkers, natriuretic peptides remain the most extensively studied in AF. Elevated concentrations of B-type natriuretic peptide (BNP) and N-terminal pro-B-type natriuretic peptide (NT-proBNP) are commonly observed in patients with persistent AF and are associated with atrial stretch, increased filling pressures, myocardial stress, and adverse cardiovascular outcomes [46,47]. Importantly, elevated natriuretic peptide levels may occur even in the absence of overt heart failure, reflecting the hemodynamic and neurohormonal consequences of AF itself.

Several studies have demonstrated significant reductions in natriuretic peptide concentrations following successful restoration of sinus rhythm [13,48]. These changes may occur within weeks after cardioversion and frequently parallel improvements in symptoms and cardiac function. Consequently, serial assessment of NT-proBNP may provide a useful adjunctive measure of myocardial recovery and reverse remodeling during follow-up.

Beyond natriuretic peptides, growing interest has focused on biomarkers associated with inflammation and fibrosis. Elevated levels of C-reactive protein, interleukin-6, galectin-3, growth differentiation factor-15 (GDF-15), and other inflammatory mediators have been linked to AF progression and adverse remodeling [26,49]. Although the clinical role of these markers remains less clearly established than that of natriuretic peptides, they may offer additional information regarding the biological substrate underlying myocardial dysfunction and recovery potential.

Cardiac troponins have also attracted attention as indicators of myocardial injury and cardiovascular risk. High-sensitivity cardiac troponin concentrations are frequently elevated in patients with AF and have been associated with adverse outcomes, including heart failure, hospitalization, stroke, and mortality [50]. However, the relationship between troponin dynamics and post-cardioversion myocardial recovery remains incompletely understood and requires further investigation.

Importantly, biomarker responses after the restoration of sinus rhythm are highly heterogeneous. Some patients demonstrate rapid normalization of biomarker profiles, whereas others exhibit persistent elevations despite the maintenance of sinus rhythm. This variability likely reflects differences in disease duration, underlying myocardial substrate, comorbidity burden, and the extent of structural remodeling. Accordingly, biomarker trajectories may provide important information regarding recovery potential and long-term prognosis.

From a translational perspective, biomarkers may serve as complementary tools within a multidimensional recovery assessment framework. The integration of biomarker dynamics with echocardiographic parameters, strain imaging, and clinical outcomes could improve the characterization of individual recovery trajectories and facilitate the identification of patients most likely to benefit from sustained rhythm-control strategies.

Although no biomarker-guided post-cardioversion management pathway currently exists, accumulating evidence supports the concept that biological recovery represents an important component of overall myocardial adaptation. Future prospective studies are needed to determine whether serial biomarker assessment can improve risk stratification and contribute to personalized management during the conceptual framework Post-Cardioversion Functional Window.

Key imaging, biomarker, and clinical indicators of myocardial recovery following the restoration of sinus rhythm are summarized in Table 3.

The evidence reviewed throughout this section demonstrates that myocardial recovery following the restoration of sinus rhythm is a multidimensional process involving structural, functional, biological, and clinical adaptation. Improvements in ventricular function, myocardial deformation parameters, biomarker profiles, symptom burden, and exercise capacity do not necessarily occur simultaneously and may follow distinct recovery trajectories among individual patients.

Importantly, the available literature suggests that successful rhythm restoration should not be viewed solely as an electrophysiological endpoint. Rather, the restoration of sinus rhythm may initiate a dynamic process of myocardial adaptation characterized by varying degrees of reverse remodeling and functional recovery. While some patients demonstrate substantial improvement across multiple domains, others exhibit persistent dysfunction despite the maintenance of sinus rhythm, indicating considerable heterogeneity in recovery potential.

This observation raises an important question: should post-cardioversion management focus exclusively on rhythm maintenance, or should it also incorporate systematic assessment of myocardial recovery? Current rhythm-control strategies primarily evaluate recurrence of AF, hospitalization, and major cardiovascular events, whereas recovery trajectories themselves are rarely incorporated into routine follow-up pathways.

The integration of imaging parameters, biomarker dynamics, and clinical outcomes may therefore provide an opportunity to move beyond traditional rhythm-based assessment and toward a more comprehensive characterization of treatment response. Such an approach could facilitate the identification of patients with active reverse remodeling, distinguish reversible myocardial dysfunction from advanced myocardial disease, and support more individualized rhythm-control management.

Collectively, these findings provide the conceptual foundation for a recovery-oriented framework of post-cardioversion care. Building upon the available evidence, we propose the concept of a Post-Cardioversion Functional Window—a period of potentially enhanced myocardial recovery during which systematic functional assessment may provide clinically meaningful information regarding treatment response, recovery potential, and long-term prognosis.

### 3.6. From Recovery Assessment to Recovery Phenotyping

The available evidence suggests that myocardial recovery following the restoration of sinus rhythm is a multidimensional process involving ventricular function, myocardial mechanics, biomarker dynamics, symptom burden, and reverse remodeling. Importantly, recovery trajectories appear highly heterogeneous, indicating that the restoration of sinus rhythm does not necessarily translate into uniform functional recovery across all patients.

Current rhythm-control strategies primarily focus on rhythm maintenance and arrhythmia recurrence, whereas systematic assessment of myocardial recovery remains limited. The integration of imaging parameters, biomarker profiles, and clinical outcomes may therefore provide a more comprehensive characterization of treatment response and identify patients with differing recovery potential.

## 4. Integrating Imaging, Biomarkers, and Clinical Recovery

The growing body of evidence reviewed in the preceding sections indicates that myocardial recovery following the restoration of sinus rhythm is a complex and multidimensional process. Improvements in ventricular function, myocardial deformation parameters, biomarker profiles, symptoms, exercise capacity, and structural remodeling collectively contribute to recovery, yet these domains are frequently assessed in isolation. As a result, current approaches may fail to fully characterize individual recovery trajectories following cardioversion.

Traditional post-cardioversion follow-up primarily focuses on the maintenance of sinus rhythm and the detection of arrhythmia recurrence. While rhythm status remains an important therapeutic endpoint, it provides limited information regarding the extent of myocardial recovery achieved after the restoration of sinus rhythm. Patients with apparently successful rhythm control may exhibit markedly different patterns of functional adaptation, ranging from substantial reverse remodeling and symptomatic improvement to persistent myocardial dysfunction despite maintenance of sinus rhythm.

Recent advances in cardiovascular imaging have significantly improved the ability to assess myocardial recovery. Conventional echocardiographic measures, including left ventricular ejection fraction and chamber dimensions, provide important information regarding global cardiac performance and structural remodeling. However, myocardial deformation imaging has demonstrated greater sensitivity for detecting early changes in myocardial function and may identify recovery before measurable changes in conventional parameters become evident [11,38,39,40,41,42,43].

Similarly, circulating biomarkers provide complementary insights into biological recovery processes that cannot be directly visualized through imaging. Natriuretic peptides reflect myocardial stress and hemodynamic burden, while inflammatory and fibrotic biomarkers may offer information regarding the underlying myocardial substrate and recovery potential [45,46,47,48,49,50]. The integration of biomarker assessment with imaging findings may therefore facilitate a more comprehensive evaluation of myocardial adaptation following cardioversion.

Importantly, clinical recovery does not always parallel structural or biological improvement. Symptom burden, exercise tolerance, quality of life, and patient-reported functional status represent essential dimensions of treatment response that may not be fully explained by imaging or laboratory parameters alone. Consequently, meaningful evaluation of recovery should extend beyond traditional cardiac measurements and incorporate patient-centered outcomes whenever possible.

Taken together, these observations support a multidimensional framework of recovery assessment in which imaging, biomarkers, and clinical outcomes are interpreted as complementary rather than competing sources of information. Such an approach may provide a more accurate representation of treatment response, improve the identification of patients with active reverse remodeling, and facilitate more individualized rhythm-control strategies.

### Rhythm Control Versus Rate Control: Remodeling, Neurohormonal Effects, and Long-Term Outcomes

Rate control remains an accepted strategy for many patients with atrial fibrillation, particularly when symptoms are limited, when rhythm-control interventions are unlikely to succeed, or when treatment burden outweighs the expected benefit. By reducing excessive ventricular rate, rate control can mitigate tachycardia-mediated injury, improve filling time, and reduce hemodynamic and neurohormonal stress. However, it does not restore atrial contraction, regular ventricular activation, or atrioventricular coordination; therefore, structural and mechanical abnormalities attributable to the persistence of AF may remain despite adequate rate control [15,16,17].

Rhythm control provides a distinct biological opportunity by restoring coordinated activation and atrial contribution to ventricular filling. In selected patients, this may be followed by reductions in chamber volumes and natriuretic peptides, improvement in left ventricular function and myocardial deformation, and partial regression of diffuse fibrosis [13,19,31,34,41,45,46,47,48,49,50]. Direct head-to-head evidence using serial morphological and neurohormonal endpoints remains limited, however, and observed recovery depends on the maintenance of sinus rhythm, disease duration, comorbidity burden, and the underlying myocardial substrate.

Evidence for hard long-term outcomes has also evolved. In the AFFIRM trial, a predominantly antiarrhythmic drug-based rhythm-control strategy did not confer a survival advantage over rate control and was associated with more hospitalizations and adverse drug effects; the RACE trial similarly demonstrated that rate control was not inferior for a composite of cardiovascular morbidity and mortality in patients with recurrent persistent AF [51,52]. By contrast, EAST-AFNET 4 showed that early rhythm control reduced a composite of cardiovascular death, stroke, and hospitalization for worsening heart failure or acute coronary syndrome compared with usual care [4]. CABANA did not demonstrate a significant reduction in its primary composite endpoint with ablation in the intention-to-treat analysis [37], whereas CASTLE-AF demonstrated lower mortality or heart-failure hospitalization with ablation in a selected population with AF and systolic heart failure [33]. These differences emphasize the importance of treatment timing, modality, patient selection, and successful rhythm maintenance.

Accordingly, the available evidence does not establish a universal superiority of rhythm control over rate control. The proposed Post-Cardioversion Functional Window is not intended to displace rate control as a valid management strategy; rather, it addresses a specific unanswered question in patients selected for cardioversion: whether and how myocardial recovery should be measured after sinus rhythm is restored. Future comparative studies should combine hard clinical endpoints with serial imaging, biomarker, rhythm-burden, and patient-reported outcomes.

Despite the increasing recognition of recovery as an important therapeutic objective, no standardized framework currently exists for the systematic assessment of myocardial recovery following cardioversion. Most contemporary rhythm-control pathways remain focused on arrhythmia recurrence rather than recovery trajectories, creating a gap between biological recovery processes and clinical decision-making. This gap provides the rationale for the development of a recovery-oriented assessment framework capable of integrating structural, functional, biological, and clinical dimensions of post-cardioversion adaptation.

Building upon the available evidence, we therefore propose the concept of a Post-Cardioversion Functional Window—a period of potentially enhanced myocardial recovery during which systematic multidimensional assessment may provide clinically meaningful information regarding treatment response, recovery potential, and long-term prognosis.

## 5. The Post-Cardioversion Functional Window: A Conceptual Framework for Myocardial Recovery Assessment

### 5.1. Conceptual Basis

Current rhythm-control strategies in atrial fibrillation primarily focus on the restoration and maintenance of sinus rhythm, the prevention of recurrence, symptom reduction, and the improvement of long-term cardiovascular outcomes [1,3]. While these objectives remain fundamental, the evidence reviewed throughout this article suggests that successful rhythm restoration may initiate a broader process of myocardial adaptation extending beyond rhythm status alone.

Accumulating data demonstrate that the restoration of sinus rhythm may be followed by improvements in ventricular function, myocardial deformation parameters, atrial mechanics, biomarker profiles, symptom burden, exercise capacity, and structural remodeling [13,31,32,33,34,35,36,37,38,39,40,41,42,43,45,46,47,48,49,50]. Importantly, these changes frequently evolve over weeks or months rather than occurring immediately after cardioversion, indicating the presence of a dynamic recovery phase characterized by ongoing biological and functional adaptation.

Despite the increasing recognition of reverse remodeling and AF-mediated cardiomyopathy, current clinical pathways rarely incorporate systematic assessment of myocardial recovery. Follow-up strategies are generally centered on rhythm surveillance, whereas the extent and trajectory of myocardial recovery are assessed inconsistently. Consequently, potentially valuable information regarding treatment response, recovery potential, and prognosis may remain underutilized in routine practice.

Based on these observations, we propose the concept of a Post-Cardioversion Functional Window (PCFW), defined as a period of potentially enhanced myocardial recovery following restoration of sinus rhythm during which serial multidimensional assessment may provide clinically meaningful information regarding myocardial adaptation and treatment response.

### 5.2. Definition of the Post-Cardioversion Functional Window

The Post-Cardioversion Functional Window is proposed as a conceptual recovery phase beginning immediately after the successful restoration of sinus rhythm and extending through the subsequent months of follow-up.

During this period, patients may experience varying degrees of

- Reverse structural remodeling;
- Recovery of ventricular mechanics;
- Recovery of atrial mechanical function;
- Normalization of biomarker profiles;
- Improvement in symptoms and functional capacity;
- Stabilization of rhythm-control outcomes.

The duration and magnitude of the conceptual framework of the functional window are likely influenced by multiple factors, including AF duration, burden of comorbidities, extent of myocardial fibrosis, baseline ventricular dysfunction, age, and maintenance of sinus rhythm. Consequently, recovery trajectories are expected to differ substantially among individual patients.

Rather than representing a fixed temporal interval, the PCFW should be viewed as a biological and functional process characterized by ongoing myocardial adaptation. In this context, serial assessment may be more informative than isolated measurements obtained at a single time point.

**Figure 4:**
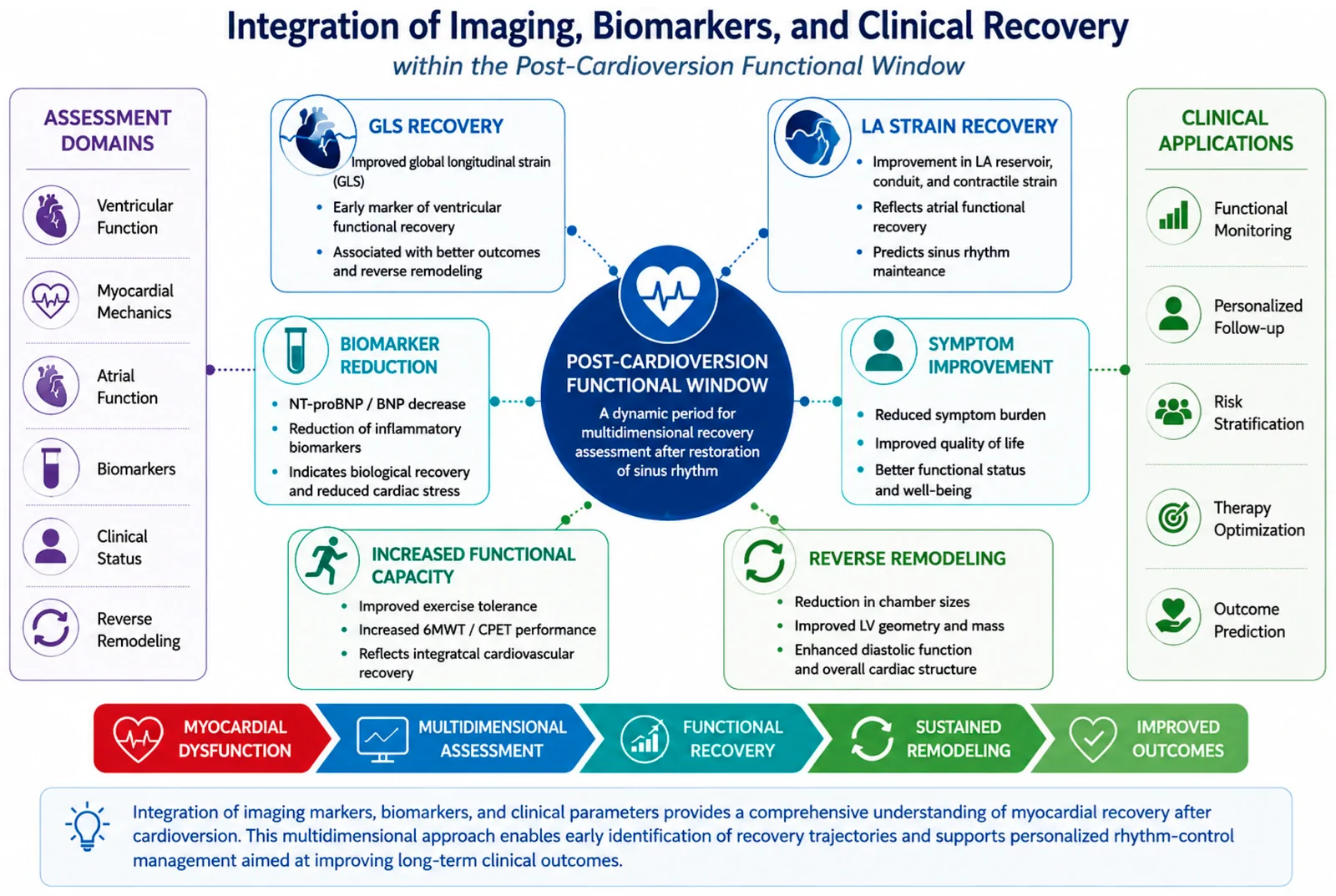
The Post-Cardioversion Functional Window: conceptual framework for multidimensional myocardial recovery assessment. The framework integrates structural, functional, biological, and clinical recovery domains and is intended as a hypothesis-generating model rather than a validated clinical pathway. Abbreviations: 6MWT, 6-min walk test; BNP, B-type natriuretic peptide; CPET, cardiopulmonary exercise testing; GLS, global longitudinal strain; LA, left atrial; LV, left ventricular; NT-proBNP, N-terminal pro-B-type natriuretic peptide.

The relationship between myocardial mechanics, biological recovery, and clinical improvement within the Post-Cardioversion Functional Window is illustrated in Figure 4.

### 5.3. Proposed Domains of Recovery Assessment

Within the conceptual framework of Post-Cardioversion Functional Window, recovery assessment may be organized into four complementary domains:

1. Structural Recovery—assessment of chamber dimensions, ventricular geometry, atrial remodeling, and reverse structural adaptation.
2. Functional Recovery—assessment of ventricular performance, myocardial deformation parameters, atrial mechanics, and exercise capacity.
3. Biological Recovery—assessment of natriuretic peptides, inflammatory biomarkers, and other indicators of myocardial stress and remodeling.
4. Clinical Recovery—assessment of symptoms, quality of life, patient-reported outcomes, functional status, and cardiovascular events.

The integration of these domains may provide a more comprehensive understanding of treatment response than rhythm status alone and may facilitate the identification of distinct recovery phenotypes following cardioversion.

### 5.4. Potential Clinical Applications

Although the conceptual framework remains conceptual, several potential clinical applications can be envisioned.

First, systematic recovery assessment may improve the characterization of treatment response beyond simple rhythm maintenance. Second, the identification of patients demonstrating active reverse remodeling may facilitate more individualized follow-up strategies. Third, multidimensional recovery assessment may support risk stratification and improve the understanding of factors associated with favorable or unfavorable recovery trajectories.

The phenotypes in Table 4 are hypothetical and require prospective validation; they illustrate how imaging, biomarker, and clinical trajectories may be integrated after cardioversion.

Importantly, the proposed conceptual framework is not intended to replace existing rhythm-control strategies but rather to complement them by incorporating recovery-oriented endpoints into post-cardioversion care.

Future prospective studies are needed to validate the concept, define optimal assessment intervals, determine clinically meaningful recovery thresholds, and evaluate whether recovery-guided management improves patient outcomes.

### 5.5. Future Directions: Toward Function-Guided Rhythm Control

Beyond demonstrating the successful restoration of sinus rhythm, the next generation of rhythm-control strategies may shift toward individualized assessment of myocardial recovery. Rather than considering cardioversion as the endpoint of treatment, future management may view it as the beginning of a dynamic recovery process requiring structured functional monitoring. Several emerging developments may facilitate this transition. First, artificial intelligence (AI) and machine learning (ML) could integrate multimodal datasets—including echocardiographic strain imaging, ECG characteristics, circulating biomarkers, and clinical variables—to predict individual recovery trajectories and identify patients most likely to benefit from prolonged rhythm-control strategies. Second, digital health technologies may enable continuous monitoring beyond rhythm surveillance alone. Wearable ECG devices, remote symptom assessment, and home-based collection of physiological parameters could be combined with scheduled imaging and biomarker evaluation to construct personalized recovery profiles over time. Third, advances in multimodal cardiac phenotyping may allow for the identification of distinct recovery endotypes following cardioversion. Rather than classifying patients solely according to the recurrence or maintenance of sinus rhythm, future studies may categorize individuals according to patterns of ventricular recovery, atrial mechanical restoration, biomarker normalization, and functional improvement. Such phenotypes could ultimately support individualized therapeutic decision-making. Finally, prospective clinical trials should evaluate whether function-guided rhythm control, in which follow-up intensity and therapeutic adjustments are informed by myocardial recovery rather than rhythm status alone, improves long-term clinical outcomes compared with conventional rhythm-based management.

If validated, the conceptual framework Post-Cardioversion Functional Window may evolve from a conceptual framework into a clinically actionable strategy for personalized management of patients with persistent atrial fibrillation.

The future of atrial fibrillation management may no longer rely exclusively on the question of whether sinus rhythm has been restored, but rather on how completely myocardial function has recovered. Moving from rhythm-guided to function-guided management may represent the next evolutionary step in personalized care for patients with persistent atrial fibrillation.

We propose the concept of *Recovery-Guided Rhythm Control*, a strategy in which serial assessment of myocardial function, myocardial deformation imaging, circulating biomarkers, and clinical recovery collectively inform post-cardioversion therapeutic decisions. Unlike conventional rhythm-guided management, this approach recognizes myocardial recovery as an independent therapeutic target. The proposed Post-Cardioversion Functional Window should be regarded as a hypothesis-generating conceptual framework intended to stimulate prospective investigation rather than an immediately applicable clinical strategy.

## 6. Toward a Myocardial Function-Guided Rhythm Control Strategy

### 6.1. Beyond Rhythm Maintenance: Redefining Treatment Success

Contemporary rhythm-control strategies in atrial fibrillation are largely evaluated according to rhythm-centered outcomes, including the maintenance of sinus rhythm, reduction in arrhythmia burden, prevention of recurrence, and improvement of major cardiovascular events [1,3]. Although these endpoints remain clinically important, they may not fully capture the biological and functional consequences of successful rhythm restoration.

The evidence reviewed throughout this article suggests that the restoration of sinus rhythm may initiate a complex process of myocardial adaptation involving reverse remodeling, the recovery of ventricular and atrial mechanics, the normalization of biomarker profiles, and improvement in clinical status. Importantly, these recovery processes may occur independently of rhythm status and may vary substantially among patients. Consequently, the maintenance of sinus rhythm alone may not adequately reflect the extent of treatment response achieved after cardioversion.

This distinction may be particularly relevant in patients with persistent AF and AF-mediated myocardial dysfunction. While some individuals experience substantial improvement in myocardial performance following the restoration of sinus rhythm, others demonstrate limited recovery despite successful rhythm maintenance. Conversely, partial recurrence of AF may occur in patients who nevertheless exhibit meaningful improvements in myocardial mechanics, biomarker profiles, and symptoms. These observations suggest that rhythm status and myocardial recovery represent related but distinct dimensions of therapeutic response.

The concept of myocardial function-guided rhythm control proposes that the assessment of treatment success should extend beyond arrhythmia recurrence and incorporate objective indicators of myocardial recovery. Such indicators may include improvements in global longitudinal strain, the recovery of atrial mechanical function, favorable biomarker dynamics, reverse remodeling, exercise capacity, and patient-reported outcomes. Together, these measures may provide a more comprehensive understanding of how individual patients respond to rhythm-control interventions.

Importantly, this proposed approach is not intended to replace established rhythm-control objectives. Rather, it complements conventional rhythm surveillance by introducing recovery-oriented endpoints capable of capturing biological and functional adaptation following the restoration of sinus rhythm. Within this framework, successful treatment may be viewed not only as maintenance of sinus rhythm but also as achievement of meaningful myocardial recovery.

From a broader perspective, the adoption of recovery-oriented endpoints may potentially facilitate a transition from a predominantly rhythm-centered model of care toward a more function-centered strategy. Such an evolution would align with contemporary trends in precision cardiovascular medicine, where treatment effectiveness is increasingly evaluated according to multidimensional outcomes rather than isolated physiological measurements.

The Post-Cardioversion Functional Window provides a potential structure through which this transition could be operationalized. By systematically evaluating myocardial recovery during the months following cardioversion, clinicians may gain additional insight into treatment response, recovery potential, and long-term prognosis beyond what can be inferred from rhythm status alone.

### 6.2. Integrating Functional Recovery into Post-Cardioversion Follow-Up

Current post-cardioversion follow-up strategies primarily focus on rhythm surveillance, symptom assessment, anticoagulation management, and the prevention of recurrent atrial fibrillation [1,3]. While these components remain essential, the evidence reviewed in previous sections suggests that myocardial recovery represents an additional and largely underexplored dimension of treatment response.

Assessment of sinus-rhythm continuity after cardioversion should be matched to the likelihood of asymptomatic recurrence and the clinical consequences of missing it. A 12-lead electrocardiogram or short Holter recording provides only a time-limited snapshot and may underestimate intermittent AF. Contemporary options include extended adhesive electrocardiographic patches, handheld or smartphone-linked single-lead electrocardiographic devices, and smartwatch-based electrocardiography or photoplethysmography, which enable repeated patient-initiated or passive measurements between clinic visits [1,2,3]. Photoplethysmographic alerts should be regarded as screening signals requiring electrocardiographic confirmation, and diagnostic yield remains dependent on wear time, adherence, signal quality, and alert burden [1,2,3].

Post-cardioversion evidence is emerging. In a single-centre retrospective implementation study of 461 acutely successful direct-current cardioversions, remote smartphone photoplethysmographic follow-up achieved high compliance, although 11.8% of monitored procedures required subsequent electrocardiography because of non-compliance, technical failure, or inconclusive recordings [53]. For selected patients in whom precise AF-burden quantification would alter rhythm-control management, continuous data from a cardiac implantable electronic device or implantable loop recorder may provide the most complete surveillance; however, invasiveness, cost, and data-management demands preclude routine use after every cardioversion [1,3]. Monitoring should therefore be individualized rather than device-driven, with greater intensity when recurrence is likely to be silent or when rhythm status would inform antiarrhythmic therapy, repeat cardioversion, or ablation planning. Monitoring results should not be used in isolation to discontinue anticoagulation, which remains guided by thromboembolic risk [1,3].

A recovery-oriented follow-up strategy would aim to evaluate not only whether sinus rhythm has been maintained but also whether the restoration of sinus rhythm has translated into meaningful improvements in myocardial structure, function, and patient well-being. Such an approach recognizes that electrical recovery and functional recovery may occur at different rates and may not always progress in parallel.

Echocardiography remains the cornerstone of functional assessment following cardioversion. Conventional parameters, including left ventricular ejection fraction, chamber dimensions, and indices of diastolic function, provide valuable information regarding reverse remodeling and global cardiac performance. However, a reliance on conventional measures alone may underestimate early stages of myocardial recovery. The integration of myocardial deformation imaging, particularly global longitudinal strain and left atrial strain, may improve the detection of subtle functional improvement and provide a more sensitive assessment of recovery trajectories [11,38,39,40,41,42,43].

Biomarker assessment may further complement imaging findings. Serial measurement of NT-proBNP, BNP, and selected markers of inflammation or myocardial stress may provide insight into biological recovery processes that precede or accompany structural adaptation [45,46,47,48,49,50]. Although routine biomarker-guided management remains investigational, the incorporation of biomarker dynamics into follow-up protocols may improve the characterization of individual recovery patterns.

Importantly, functional recovery should not be evaluated exclusively through imaging and laboratory parameters. Symptoms, exercise tolerance, quality of life, and patient-reported outcomes represent clinically meaningful dimensions of treatment response and may provide unique information regarding the real-world impact of rhythm-control interventions. The integration of patient-centered outcomes into recovery assessment would align with contemporary value-based and personalized care models.

Within the conceptual framework of the Post-Cardioversion Functional Window, serial multidimensional assessment may potentially facilitate the identification of distinct recovery trajectories. Patients demonstrating progressive improvement across multiple domains may represent a favorable recovery phenotype, whereas persistent abnormalities despite the maintenance of sinus rhythm may identify individuals requiring closer monitoring, optimization of comorbidity management, or alternative therapeutic strategies.

Although the optimal timing of recovery assessment remains to be established, available evidence suggests that clinically meaningful changes may occur during the first several months following cardioversion. Consequently, structured reassessment at predefined intervals may provide a practical framework for monitoring myocardial adaptation and informing individualized clinical decision-making.

A proposed recovery-oriented follow-up pathway integrating imaging, biomarkers, and clinical assessment is presented in Table 5.

The proposed pathway is intended as a conceptual framework rather than a prescriptive clinical algorithm. Its purpose is to illustrate how recovery-oriented assessment could be integrated into contemporary rhythm-control management and to provide a foundation for future prospective validation studies.

### 6.3. Future Validation of the Post-Cardioversion Functional Window

The Post-Cardioversion Functional Window is proposed as a hypothesis-generating framework intended to advance the understanding of myocardial recovery following the restoration of sinus rhythm. Although current evidence supports the existence of reverse remodeling, functional improvement, and biomarker normalization after successful rhythm restoration, these processes have largely been investigated as isolated phenomena rather than components of an integrated recovery pathway.

Prospective validation of the proposed framework represents an important next step. Future studies should determine whether distinct recovery trajectories can be identified reproducibly and whether multidimensional assessment provides clinically meaningful information beyond conventional rhythm monitoring. Particular attention should be given to the temporal evolution of recovery, as available evidence suggests that structural, functional, biological, and clinical adaptation may occur at different rates following cardioversion.

Several key research questions remain unanswered. First, the optimal duration of the conceptual frameworks of the functional window has not been established. While improvements in myocardial mechanics and biomarker profiles have been reported during the first months after the restoration of sinus rhythm, the period during which recovery remains most dynamic is unknown. Second, clinically meaningful thresholds for recovery have not been defined. Future investigations should determine whether changes in parameters such as global longitudinal strain, atrial strain, natriuretic peptides, or patient-reported outcomes can identify patients with favorable recovery trajectories and improved long-term prognosis.

Another important area of investigation is the characterization of recovery phenotypes. It is plausible that patients with persistent AF demonstrate heterogeneous patterns of adaptation, ranging from complete reverse remodeling and functional normalization to persistent myocardial dysfunction despite the maintenance of sinus rhythm. The identification of such phenotypes could facilitate risk stratification and contribute to more personalized rhythm-control strategies.

The integration of patient-reported outcomes represents an additional opportunity for future research. While recovery is often evaluated through imaging and biomarker assessment, improvements in symptoms, functional capacity, and quality of life may represent equally important dimensions of treatment success. Incorporating patient-centered measures into recovery assessment frameworks would align with contemporary value-based healthcare principles and may provide a more comprehensive understanding of treatment response.

Emerging technologies may further enhance recovery assessment. Wearable devices, remote rhythm monitoring, artificial intelligence-assisted echocardiographic analysis, and digital health platforms have the potential to enable continuous characterization of recovery trajectories beyond traditional clinic-based follow-up. Such approaches could facilitate earlier detection of unfavorable recovery patterns and support more individualized patient management.

Ultimately, validation of the Post-Cardioversion Functional Window will require prospective longitudinal studies incorporating serial imaging, biomarker assessment, and clinical outcomes. If confirmed, the proposed conceptual framework may contribute to a transition from rhythm-centered evaluation toward a more comprehensive recovery-oriented model of care in patients with persistent atrial fibrillation.

## 7. Conclusions

Persistent atrial fibrillation is associated with complex electrical, structural, mechanical, and biological remodeling processes that contribute to myocardial dysfunction and adverse clinical outcomes. Increasing evidence indicates that the restoration of sinus rhythm may initiate reverse remodeling and functional recovery involving ventricular performance, atrial mechanics, biomarker profiles, symptom burden, and overall cardiovascular status.

Current rhythm-control strategies primarily focus on the maintenance of sinus rhythm and prevention of arrhythmia recurrence. However, the available literature suggests that rhythm restoration may represent only one component of treatment success. Recovery trajectories appear heterogeneous and may provide clinically meaningful information regarding myocardial reversibility, treatment response, and long-term prognosis.

Advances in echocardiography, myocardial deformation imaging, biomarker assessment, and patient-centered outcome evaluation have created new opportunities to characterize myocardial recovery beyond conventional rhythm-based endpoints. Integration of these complementary domains may facilitate a more comprehensive understanding of post-cardioversion adaptation and improve the identification of patients with favorable or unfavorable recovery trajectories.

Building upon the current evidence, we propose the concept of a Post-Cardioversion Functional Window—a period of potentially enhanced myocardial recovery following the restoration of sinus rhythm during which systematic multidimensional assessment may provide valuable information regarding reverse remodeling, recovery potential, and clinical outcomes. Although the proposed conceptual framework remains hypothesis-generating, it offers a foundation for future prospective investigation and may support the development of recovery-oriented rhythm-control strategies.

Future research should focus on validating recovery trajectories, defining recovery phenotypes, and determining whether myocardial function-guided management can improve outcomes beyond conventional rhythm monitoring. Such an approach may contribute to a transition from rhythm-centered evaluation toward a more comprehensive, function-centered model of care for patients with persistent atrial fibrillation.

## Figures and Tables

**Table 1:** Principal mechanisms of myocardial dysfunction in persistent atrial fibrillation.

Mechanism	Pathophysiological Effect	Clinical Consequences
Irregular ventricular rhythm	Beat-to-beat variability, impaired hemodynamics	Reduced cardiac output
Tachycardia-mediated injury	Increased myocardial oxygen demand, calcium dysregulation	LV dysfunction, cardiomyopathy
Loss of atrial contraction	Reduced ventricular filling	Exercise intolerance
Structural remodeling	Chamber dilation, fibrosis	Progressive AF maintenance
Neurohormonal activation	RAAS and sympathetic activation	Heart failure progression
Inflammation	Cytokine-mediated myocardial injury	Fibrosis and remodeling
Mechanical dysfunction	Reduced atrial and ventricular strain	Functional impairment
Comorbidity burden	Hypertension, obesity, diabetes	Accelerated remodeling

Abbreviations: AF, atrial fibrillation; LV, left ventricular; RAAS, renin–angiotensin–aldosterone system.

**Table 2:** Biological mechanisms and potential markers of myocardial dysfunction and recovery in persistent AF.

Mechanism	Principal Pathophysiology	Potential Markers	Potential Recovery Indicator
Neurohormonal activation	RAAS and sympathetic activation	NT-proBNP, BNP	Reduction in natriuretic peptides
Inflammation	Cytokine-mediated myocardial injury	CRP, IL-6	Reduction in inflammatory burden
Fibrosis	Extracellular matrix expansion	LA size, MRI fibrosis markers	Reverse remodeling
Mechanical dysfunction	Impaired myocardial deformation	GLS, atrial strain	Improved strain parameters
Hemodynamic impairment	Reduced filling and cardiac output	Functional status	Improved exercise capacity
AF-mediated cardiomyopathy	Ventricular dysfunction	LVEF, GLS	Recovery of ventricular function

Abbreviations: AF, atrial fibrillation; BNP, B-type natriuretic peptide; CRP, C-reactive protein; GLS, global longitudinal strain; IL-6, interleukin-6; LA, left atrial; LVEF, left ventricular ejection fraction; MRI, magnetic resonance imaging; NT-proBNP, N-terminal pro-B-type natriuretic peptide; RAAS, renin–angiotensin–aldosterone system.

**Table 3:** Proposed indicators of myocardial recovery within the Post-Cardioversion Functional Window.

Domain	Marker	Expected Recovery Pattern	Clinical Relevance
Ventricular function	LVEF	Increase	Recovery of systolic function
Myocardial mechanics	GLS	Improvement	Early reverse remodeling
Atrial mechanics	LA strain	Improvement	Atrial functional recovery
Biomarkers	NT-proBNP	Reduction	Reduced myocardial stress
Biomarkers	BNP	Reduction	Hemodynamic improvement
Biomarkers	CRP, IL-6	Reduction	Reduced inflammatory burden
Clinical status	Symptoms	Improvement	Patient-perceived recovery
Functional status	Exercise capacity	Improvement	Functional recovery
Structural recovery	Chamber volumes	Reduction	Reverse remodeling

Abbreviations: BNP, B-type natriuretic peptide; CRP, C-reactive protein; GLS, global longitudinal strain; IL-6, interleukin-6; LA, left atrial; LVEF, left ventricular ejection fraction; NT-proBNP, N-terminal pro-B-type natriuretic peptide.

**Table 4:** Proposed recovery phenotypes within the Post-Cardioversion Functional Window.

Phenotype	Imaging	Biomarkers	Symptoms	Expected Prognosis
Complete Recovery	GLS ↑, LA strain ↑	NT-proBNP ↓	Major improvement	Favorable
Predominantly Functional Recovery	GLS ↑	Mild biomarker change	Improvement	Intermediate
Predominantly Biological Recovery	Biomarkers ↓	Minimal imaging change	Variable	Intermediate
Limited Recovery	Minimal changes	Persistent elevation	Persistent symptoms	Higher risk

Abbreviations: GLS, global longitudinal strain; LA, left atrial; NT-proBNP, N-terminal pro-B-type natriuretic peptide.

**Table 5:** Proposed recovery-oriented follow-up pathway within the Post-Cardioversion Functional Window.

Timepoint	Rhythm Assessment	Imaging Assessment	Biomarkers	Clinical Assessment
Baseline (pre-cardioversion)	12-lead ECG	Echocardiography (LVEF, GLS, LA strain); CMR if indicated	NT-proBNP, BNP	Symptoms, QoL
1 Month	12-lead ECG ± patch/wearable monitoring	GLS, LA strain	NT-proBNP	Symptoms
3 Months	12-lead ECG ± patch/wearable monitoring	Echocardiography (LVEF, GLS, LA strain)	NT-proBNP	Symptoms, exercise capacity
6 Months	Risk-adapted ECG monitoring	Comprehensive echocardiography; CMR if indicated	NT-proBNP ± additional biomarkers	Symptoms, QoL, clinical outcomes

Abbreviations: BNP, B-type natriuretic peptide; CMR, cardiac magnetic resonance; ECG, electrocardiography; GLS, global longitudinal strain; LA, left atrial; LVEF, left ventricular ejection fraction; NT-proBNP, N-terminal pro-B-type natriuretic peptide; QoL, quality of life.

## Data Availability

No new data were created or analyzed in this study. Data sharing is not applicable to this article.

## Abbreviations

The following abbreviations are used in this manuscript:

ACC	American College of Cardiology
ACCP	American College of Chest Physicians
AF	Atrial Fibrillation
AF-CARE	Atrial Fibrillation–CARE pathway
AI	Artificial Intelligence
AiCM	Arrhythmia-Induced Cardiomyopathy
BNP	B-type Natriuretic Peptide
CCT	Cardiac Computed Tomography
CMR	Cardiac Magnetic Resonance
CRP	C-Reactive Protein
ECG	Electrocardiography
ESC	European Society of Cardiology
GDF-15	Growth Differentiation Factor-15
GLS	Global Longitudinal Strain
HF	Heart Failure
HRS	Heart Rhythm Society
IL-6	Interleukin-6
LA	Left Atrium / Left Atrial
LA strain	Left Atrial Strain
LGE	Late Gadolinium Enhancement
LV	Left Ventricle / Left Ventricular
LVEF	Left Ventricular Ejection Fraction
ML	Machine Learning
NT-proBNP	N-terminal pro-B-type Natriuretic Peptide
PCFW	Post-Cardioversion Functional Window
PET	Positron Emission Tomography
RAAS	Renin–Angiotensin–Aldosterone System
RGRC	Recovery-Guided Rhythm Control
SPECT	Single-Photon Emission Computed Tomography
